# Experimental and predictive analysis of deep eutectic solvent gel membranes for efficient CO_2_ separation

**DOI:** 10.1038/s41598-025-14520-z

**Published:** 2025-08-13

**Authors:** Remya Ranjith, Bharti Saini, Swapnil Dharaskar, Tushar Patil, Grishma Pindolia, Satyam Shinde, Rama Rao Karri

**Affiliations:** 1 Department of Chemical Engineering, School of Energy Technology, Pandit Deendayal Energy University, Gandhinagar, Gujarat 382426 India; 2 Department of Physics, School of Energy Technology, Pandit Deendayal Energy University, Gandhinagar, Gujarat 382426 India; 3 Chemical and Energy Engineering, Faculty of Engineering, Universiti Teknologi Brunei, Bandar Seri Begawan, BE1410 Brunei Darussalam

**Keywords:** Deep eutectic solvents, DES-gel membranes, CO_2_ separation, Permeability, Membrane separation, Density functional theory, Environmental chemistry, Environmental impact

## Abstract

The capture of Carbon Dioxide (CO_2_) is very relevant nowadays as global warming hits its peak. The separation of CO_2_ using membranes has received wide recognition by researchers because of its energy efficiency. Various Ionic Liquid supported membranes have been proven effective in this regard; however, their higher cost and toxicity are limitations, which opens possibilities for Deep Eutectic Solvents (DES). This work explains how DES gel membrane fabrication separates CO_2_ from CO_2_/CH_4_ mixtures. DES, composed of choline chloride and glycerol, is mixed with Pebax1657 polymer, and Polyvinylidene fluoride sheets are used as supports for casting. Fourier transform infrared spectroscopy has been used to confirm the synthesis of DES. X-ray Diffraction and Scanning Electron Microscopy analysis were used to analyse the membrane structure and cross-section. The physicochemical properties of DES are measured at a temperature range from 293.15 to 343.15 K. Pure and mixed gas permeabilities of CO_2_ and CH_4_ with increased pressure have been calculated. The highest permeability values obtained for pure and mixed gas CO_2_ were 138.98 Barrer and 93.17 Barrer, respectively. Density Functional Theory (DFT) is also applied to predict the interaction energy between DES and gas molecules. The efficacy of the DES-gel membrane was evaluated against other DES-supported liquid Membranes, revealing that DES may serve as a viable substitute for hazardous and costly ionic liquids.

**Supplementary Information:**

The online version contains supplementary material available at 10.1038/s41598-025-14520-z.

## Introduction

The influence of greenhouse gases generated by human activities on global warming and its irreversible environmental effects is extensively documented and has been a focal point of rigorous global discussions for several decades^1,2^. The Inter-Government Panel for Climate Change (IPCC) advocates for a 2 °C decrease in the average atmospheric temperature to avert catastrophic outcomes, proposing that carbon dioxide (CO_2_) concentrations be maintained below 450 ppm by the year 2100^3^ as a feasible solution. Carbon Capture and Storage (CCS) is a comprehensive term for technologies that mitigate CO_2_ emissions at the source by isolating CO_2_ from other combustion by-products in industrial flue gas and transporting it to storage locations, such as depleted underground reservoirs^4^. Its adoption has increased in recent years due to its potential to meet the significant objectives established by IPCC experts^5^.

In response to ongoing global warming, efforts are being made to explore innovative technologies to mitigate CO_2_ emissions^6^. CO_2_ is a prominent greenhouse gas, alongside water vapour, methane, volatile organic compounds, nitrous oxide, and ozone^1,7^. The primary technique suggested for this objective is CCS. CCS technology includes the separation of CO_2_, the process of separating CO_2_ from flue gases, using membranes^8,9^. Solvents used for the fabrication of membranes include Ionic Liquids (IL)^10,11^ and Deep Eutectic Solvents (DES)^12^. Although ILs have proved to be an effective solvent for CO_2_ applications, they also have drawbacks^13^. A significant issue is their elevated viscosity, adversely impacting the mass flow rate and permeability.

Furthermore, the synthesis is intricate and necessitates non-biodegradable and costly toxic precursors that render them commercially unsuitable for numerous applications^14^. So, DES, also a subclass of ILs that is used as an alternative to ILs^15,16^. DES are a mixture of hydrogen bond acceptor (HBA) and hydrogen bond donor (HBD)^17^. Simple mixing and stirring make their synthesis easier^18^.

Extensive research has been conducted on the separation of CO_2_ using membranes supported by DES^19,20^. Almost all the research on DES-supported membranes includes temperature dependency on the membrane’s permeability. Many researchers have used ChCl as HBA. ChCl’s capacity to create adjustable, economical, and environmentally benign DES with significant CO₂ affinity renders it an exceptional hydrogen bond acceptor for gas separation applications. Its efficacy competes with traditional solvents while mitigating critical drawbacks such as toxicity and energy-demanding regeneration.

DES-supported membranes for CO_2_ separation were put into use more than a decade ago. Some of the relevant work done specifically related to DES-supported membranes is detailed here. Amira et al.^21^ conducted the first study on the CO_2_ permeability of a PVDF (Polyvinylidene fluoride)-Co PTFE (Polytetrafluoroethylene) membrane. This membrane has DES-filled pores consisting of choline chloride and urea. The synthesis technique employed for the membrane was phase inversion. The permeability values were nearly twice as high as the empty membrane’s (25.5 × 10^3^ GPU). Ishaq et al.^22^ in their study used theoretical and experimental methods to examine the CO_2_ permeability of Choline Chloride (ChCl)/Urea (varying molar ratios) DES with PVDF membrane as a porous support. The prepared DES membranes underwent testing to determine their permeability and selectivity for pure and mixed gas streams. At a molar ratio of 2:1, DES exhibited the most outstanding permeability of 45.6 Barrer, with CO_2_/CH_4_ and CO_2_/N_2_ selectivity of 61.62 and 78.62, respectively. Ishaq et al.^23^ in their study on DES membranes investigated the use of three distinct alkanol amines as the HBD and choline chloride as the HBA. Porous PVDF was used as the support. The membranes produced exhibited a selectivity of 70.47 for CO_2_/CH_4_ and 78.86 for CO_2_/N_2_. In another study conducted by Lian et al.^24^, they used an amino acid-based DES consisting of L-arginine and ethylene glycol. They integrated this DES into the Pebax 1657 polymer to produce membranes. Compared to the pure Pebax membrane, their selectivity increased by 17%. Saeed et al.^25^ in their study used betain-based DES in conjunction with PVDF support to determine the permeability of CO_2_ from a CO_2_/CH_4_ mixture. The obtained values reached a maximum of 29.33 Barrer, and the selectivity reached 56.4. These are the studies conducted in DES on membranes for CO_2_ separation. A recent study by Nowosielski et al.^26^ used ChCl and its derivatives as HBAs and 1,2-propanediol as HBD. They prepared supported liquid membranes (SLM) with DES, and the permeability and selectivity were calculated for CO_2_ gas. Craveiro et al. ^27^ also used ChCl-urea-based DES along with a PTFE membrane and reported the highest permeability value for CO_2_ gas.

In this study, ChCl-glycerol DES, in combination with Pebax 1657 polymer, has been used to separate CO_2_ from the CO_2_/CH_4_ mixture. The innovation of the study involves the amalgamation of ChCl–Glycerol DES and Pebax1657 polymer. In nearly all published research, the fabrication of DES-supported membranes employs the pore-filling method. However, DES is uniformly distributed within the membrane matrix, which facilitates the use of a minimal quantity of solvent for casting. Further, dependency on permeability with pressure was also investigated. The DES is dissolved uniformly in the polymer solution to make a homogeneous mixture, which forms a DES gel membrane. The DES is trapped in the polymer matrix, which gives a uniform distribution of DES along the membrane surface. DES was prepared by mixing ChCl and glycerol in the ratio of 1:2. Fourier Transform Infrared Spectroscopy (FTIR) and Thermogravimetry (TGA) were studied to confirm the synthesis of DES. The dependency of Permeability and selectivity of CO_2_ gas with an increase in transmembrane pressure has been established. Detailed DFT (Density Functional Theory) studies were carried out to calculate the interactive energies of DES with component gases and to predict whether their interaction type was either physical or chemical.

## Experimental section

### Materials & chemicals

DES is synthesized using ChCl and Glycerol; PVDF sheet, Pebax 1657 (Polyether block amide), ethanol, and DI water were used for polymer casting solutions. Chemicals were purchased from Sigma-Aldrich with ≥ 99% purity. Table 1 indicates the details of chemicals, structure, and materials.

**Table 1:** Materials and chemicals with purity and molecular structure.

Chemicals	Specification	Structure
Choline chloride	Sigma Aldrich ≥ 99%	
Glycerol	Sigma Aldrich ≥ 99%	
Polyvinylidene fluoride (PVDF)	Sigma Aldrich Pore size 0.45 µm	
Pebax1657 (Polyether block amide)	60 wt% polyethylene oxide and 40 wt% polyamide Arkema speciality polyamides	
Ethanol	Sigma Aldrich purity ≥ 99.9%	

### Preparation of DES

The DES was prepared by mixing mixing-stirring method. ChCl as HBA and glycerol as HBD were taken in the molar ratio 1:2 (weighed appropriate mass in a Mettler Toledo balance with a precision of 0.00001 g). Components are mixed in a magnetic stirrer at 50 °C until they form a clear homogeneous liquid without any precipitate. The obtained DES was a stable colorless liquid at room temperature, kept in tight bottles to prevent contamination.

### Physico-chemical properties of DES

Viscosity and Density values are measured using a Thermoscientific Haake Viscotester IQ rheometer and Anton Paar DMA 4500 M Density meter at a temperature range of 20–70 °C. The temperature of the Rheometer samples was controlled using a thermostatic water bath with a precision of ± 0.01 K.

### Moisture content analysis

Moisture content of the DES and prepared DES gel membranes is determined using the Karl-Fischer titration method using ANALAB Karl Fischer Titrator µAquaCal 100 Model. The resulting value of water content results from an average of at least three different measurements.

### Synthesis of membrane

The DES-gel membrane was cast using solution casting. Initially, 15% Pebax (Poly-ether-block-amides) 1657 is combined with solvents (ethanol–water in a 70:30 ratio) and stirred thoroughly on a magnetic stirrer at 50 °C until the polymer melts. After the polymer has melted into the homogeneous solution, varying concentrations of DES are introduced, like 15 wt%, 25 wt%, and 35 wt%, and the mixture continues stirring for an additional hour. The polymer solution is applied to a porous PVDF (Polyvinylidene fluoride) sheet using a casting knife. The thickness of the casting film set was 250 µm. The prepared membrane was subsequently placed overnight in a hot air oven for solvent evaporation. Notations used to indicate the fabricated membrane are abbreviated in Table 2.

**Table 2:** Fabricated membrane notations.

Sl. No	DES wt% (based on polymer weight)	Abbreviation
1	0	Neat Pebax
2	15	GLY-15
3	25	GLY-25
4	35	GLY-35

### Fourier transform infrared spectroscopy (FTIR)

FTIR for membrane and DES were performed using a PerkinElmer Incorporated, Germany, for a wavenumber range of 4000–400 cm^−1^. All the respective functional groups and vibrations in bonds present in both membranes and DES were analysed. All the analyses were performed at room temperature.

### Thermogravimetric analysis (TGA)

TGA of the DES sample has been done using Netzsch DSC 204 F1 Phoenix in a nitrogen gas environment at a flow rate of 200 ml/min. The temperature range was 35–650 °C at a scan rate of 10 °C/min. Both heating and cooling cycle operations have been performed to analyze the peaks.

### Scanning electron microscopy (SEM)

The Field Emission Scanning Electron Microscopy (Zeiss Ultra55) was utilised to analyse the morphology of the top surface and the membrane cross-section. Before the examination, membrane samples underwent a coating process involving a thin layer of gold with a specified layer thickness of 5 nm.

### Gas permeability measurement

**Fig. 1:**
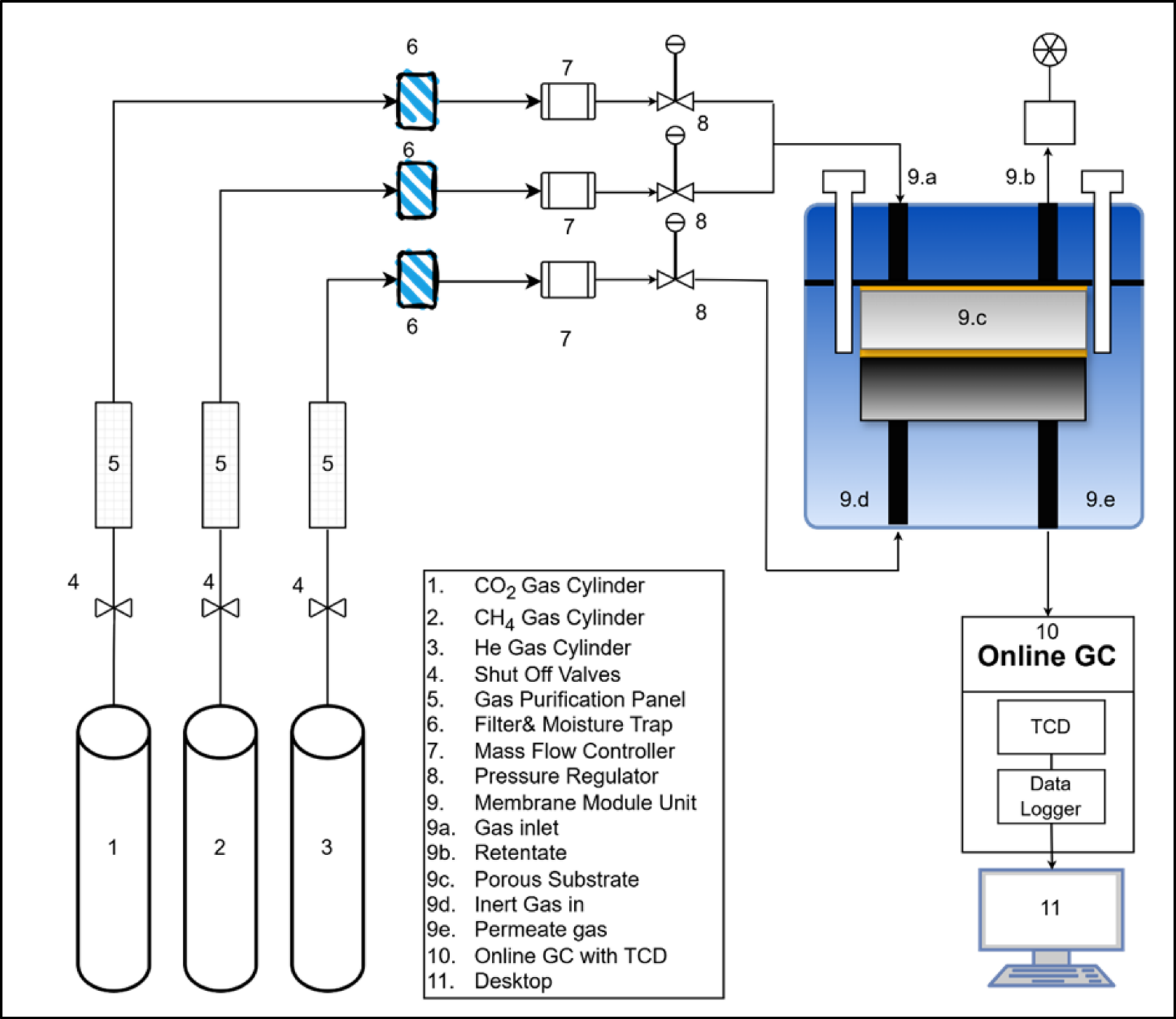
Schematic diagram of experimental setup.

All gas permeability experiments are conducted utilizing the apparatus depicted in Fig. 1. All interconnected sections and components of the setup are accurately labelled. Three gas cylinders containing CO_2_, CH_4_, and helium are of the utmost purity. All components of the setup are interconnected via 0.25-inch wires and pipelines. The gas from the cylinders initially traverses gas purification panels and a moisture trap, which are directly linked to the cylinders, as illustrated in Fig. 1. The gases subsequently traverse the mass flow controllers (MFC) (manufactured by Aalborg Instruments & Controls, Inc. (USA)—model No. GFC-17 Series), utilised to regulate the volumetric flow rates of gases. Each gas possesses a specific MFC with varying flow rate ranges. Helium exhibits a mass flow range of 0–50 ml/min, while CO_2_ and CH_4_ have flow ranges of 0–500 ml/min. All MFCs possess a standard accuracy of ± 1% of full scale. At an adjusted flow rate, gases from the MFC are directed to the membrane module unit (Fig. 1-part 9), which contains a flat sheet membrane.

**Fig. 2:**
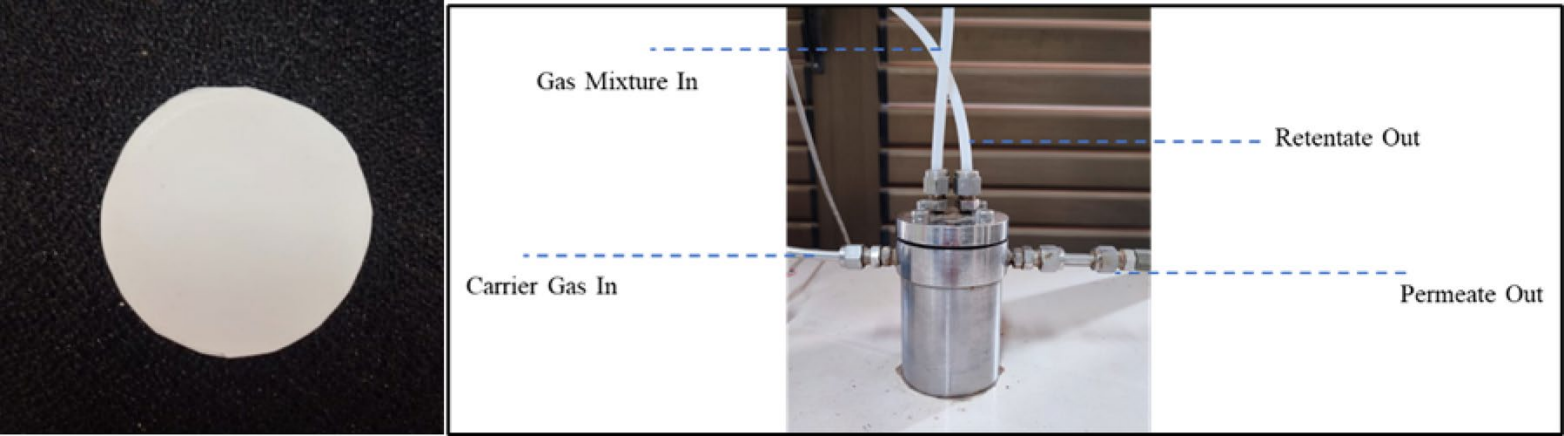
(**a**) Membrane cut; (**b**) Flat-sheet membrane module.

**Fig. 3:**
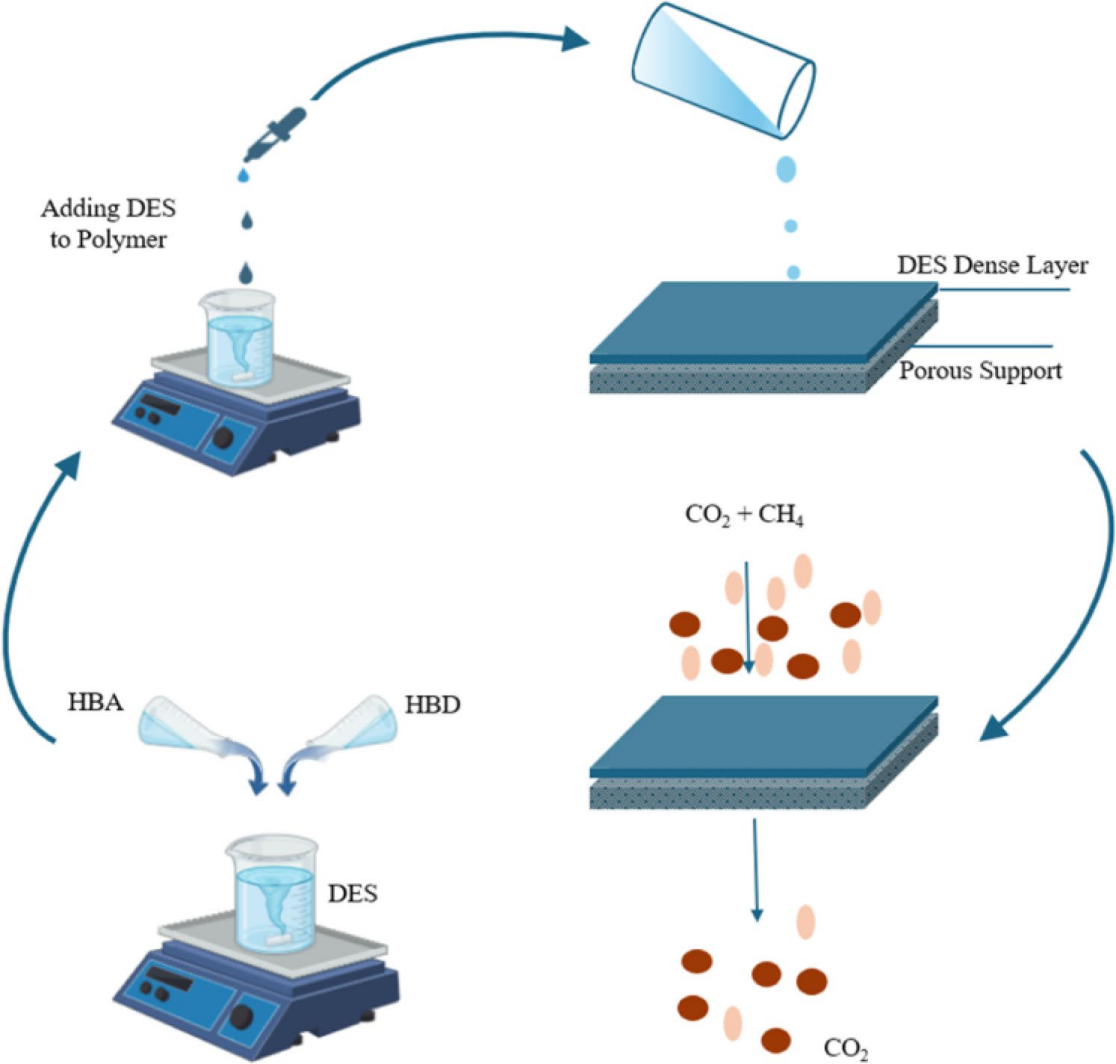
Schematic overview of the deep eutectic solvent gel membrane for efficient CO_2_ (Partly created in BioRender. Ranjith, R. (2025) https://BioRender.com/4eqhhwe).

A magnified image of the membrane module and the gas passage routes included with the setup are shown in Fig. 2. The membrane module features a ceramic porous substrate that supports the flat sheet membrane. A transmembrane pressure gauge is positioned between the permeate and retentate sides of the membrane. The module has been adequately sealed with two O-shaped gaskets, preventing gas leakage. All pressure indicators are procured from WIKA Alexander Wiegand SE & Co. KG (Germany). The back pressure regulators are acquired from Emerson Electrical Co. (US), Model TESCOM 44-2363-24. The composition of permeate gases is analysed using a gas chromatograph (Nucon India Ltd, serial no. 5700), which is directly linked to the permeate line via a six-way valve. The gas chromatograph is linked to a thermal conductivity detector (TCD) directly connected to data analysis software. All measurements are conducted in a moisture-free environment and repeated three times for consistency. The overview of the deep eutectic solvent gel membranes for efficient CO_2_ separation applications is shown in Fig. 3.

Figure S1 shows the actual experimental setup with the cut membrane that is used for testing. The gas permeability for both pure and mixed gases is measured at four different transmembrane pressures. The pressure inside the flat sheet membrane is adjusted using the outlet valve. The Gas chromatograph connected to the setup has a 6-way valve, which helps to inject the same volume of gas each time into the chromatograph. Experiments are repeated three times with each pressure. After drying, the membranes are put in a sealed bag and kept inside the vacuum desiccator to avoid any moisture retention. Since the membranes are cast using a digital casting knife, the thickness of a selective layer of Pebax 1657 is expected to be uniform throughout the membrane. The area of the membrane is used to calculate permeability. The thickness of the membrane is calculated from the SEM cross-section of the membrane. Each concentration wt% membrane follows the same procedure. Gas leakage through the membrane module is sealed properly through rubber “o” rings. The permeability is calculated using the formula given below.

$$P = \frac{{Q_{i} L}}{{\Delta P_{i} A}}$$

$$\Delta P = P_{1} - P_{2}$$

where P is the permeability (Barrer); Q is the volumetric flow rate of gas (ml/s); L is the thickness of the membrane (cm); A is the effective area of the membrane (cm^2^); $\Delta P$ is a pressure across the membrane; P_1_ is the feed side pressure, and P_2_ is the permeate side pressure.

The ratio of pure gas permeabilities may be used to calculate the ideal selectivity, indicated by S: $S = \frac{{P_{{{\text{CO}}_{2} }} }}{{P_{{{\text{CH}}_{4} }} }}$.

#### Computational methodology

Density Functional Theory (DFT) calculations were employed to elucidate the molecular-level interactions between the DES and gas molecules (CO_2_ and CH_4_) within the gel membrane matrix. The interaction energies were computed to quantify the affinity of the DES toward each gas species. The results revealed that the DES exhibits a more favourable and stronger interaction with CO_2_ than with CH_4_, as evidenced by the significantly more negative interaction energy values for the DES–CO_2_ complex. This suggests a higher thermodynamic stability and stronger binding of CO_2_ within the DES environment. To further visualize and understand the nature of these interactions, reduced density gradient (RDG) and non-covalent interaction (NCI) analyses were performed. The NCI plots revealed pronounced green-colored isosurfaces in the DES–CO_2_ system, indicative of dominant van der Waals and hydrogen bonding interactions, while the DES–CH_4_ complex showed comparatively weaker and more dispersed interactions. These findings corroborate the experimental results of higher CO_2_ solubility and selectivity and confirm that the enhanced performance of the DES-based membrane is governed by preferential non-covalent interactions with CO_2_ over CH_4_ at the molecular level.

Gaussian09 software^28^ has been utilized to implement DFT. The molecular structures of the gases (CO_2_/CH_4_), ChCl/glycerol (DES), and their complexes have been optimized using the B3LYP^29^ functional in conjunction with the 6-311G (d,p) basis set. Frequency calculations have also been carried out to ascertain the local minima of the optimized structures in the potential energy surface (PES). The basis set superimposition errors have been dealt with using counterpoise correction^30^. All computational details, including optimized structures, interaction energies, and NCI plots, are provided in the Supplementary Information^31–33^ (Figs. S3–S8). The stabilization energy of DES with CO_2_ and CH_4_ is given in Table 3.

**Table 3:** Stabilization energies for most significant donor–acceptor interactions.

Complex	Donor	Acceptor	E_2_ (kcal/mol)
DES-CO_2_	LP (O38)	σ* (C37–O39)	112.17
DES-CH_4_	LP (Cl22)	σ* (O24–H35)	15.98

## Results and discussion

### Characterization of DES using FTIR, TGA, and determination of physico-chemical properties and moisture content analysis

Knowledge of the physicochemical properties of the solvent, including its viscosity, density, refractive index, and conductivity, is essential for analysing gas transport. Density at different temperatures is measured and presented in Table 4. With an increase in temperature, density is found to decrease. DES densities are higher than those of its individual components, which can be explained by hole theory. Mixing DES components reduces the average hole’s radius and thus increases DES density relative to that of the individual constituents^34^. The amount of HBD also affects the DES values. When the amount of HBD increases, the density of DES decreases. In DES, based on ChCl as HBA and glycerol as HBD, with an increasing amount of HBD density, it was found to decrease^35^. Viscosity also has the same effect as increasing temperature. At 20 °C, the viscosity is found to be 341 mPaS, and it keeps on decreasing with increasing temperature, and it is in good accordance with the literature^36,37^. Viscosity is crucial as it affects the mass transfer and diffusion of gas through the solvent. The refractive index value indicates a component’s purity and concentration.

**Table 4:** Physico-chemical properties of DES.

Temperature (°C)	Density (g/cc)	Viscosity (mPaS)	Refractive index	Conductivity mS/cm
20	1.193	341	(measured at RT)^a^	(Measured at RT)^a^
30	1.188	176	1.486	1.7
40	1.182	100		
50	1.176	62		
60	1.171	41		
70	1.165	28.2		

^a^Measured temperature at 25 °C.

The values of moisture content are tabulated in the given tables separately for DES and DES gel membranes in Tables 5 and 6. All the Karl Fischer titrations are repeated three times, and the average is written in the table. For calculating the moisture content of DES gel membranes, first, the membrane is dissolved in N-methyl-2-pyrrolidone (NMP) using a magnetic stirrer. A specific cut of membrane (1 cm × 1 cm) has been dissolved in 15 ml NMP and used for Karl Fischer Titration.

**Table 5:** Moisture content of DES.

DES	Molar ratio	Moisture content (wt%)
Choline chloride: glycerol	1:2	0.71

**Table 6:** Moisture content of DES gel membranes.

Membrane	DES concentration (wt%)	Moisture content (wt%)
Neat Pebax	0	0.9553
GLY-15	15	1.0149
GLY-25	25	0.8644
GLY-35	35	0.9461

The result of the analysis indicates that the presence of moisture is less in the membrane. The presence of water content in the membrane is controlled by keeping the membrane in the oven for overnight drying before putting it into the flat sheet module of the experimental setup. The effect of water on DES properties and its gas transport performance is explained by Kun Xin et al.^38^. In their research, the authors experienced a decrease in the performance of supported liquid membrane with an increase in water content, which was contrary to their expectations, as higher water content decreases the viscosity of DES, which further increases the gas transfer. At higher water content, the loss of structural cohesiveness can happen in the membrane matrix and, in turn, make the fluid phase heterogeneous.

**Fig. 4:**
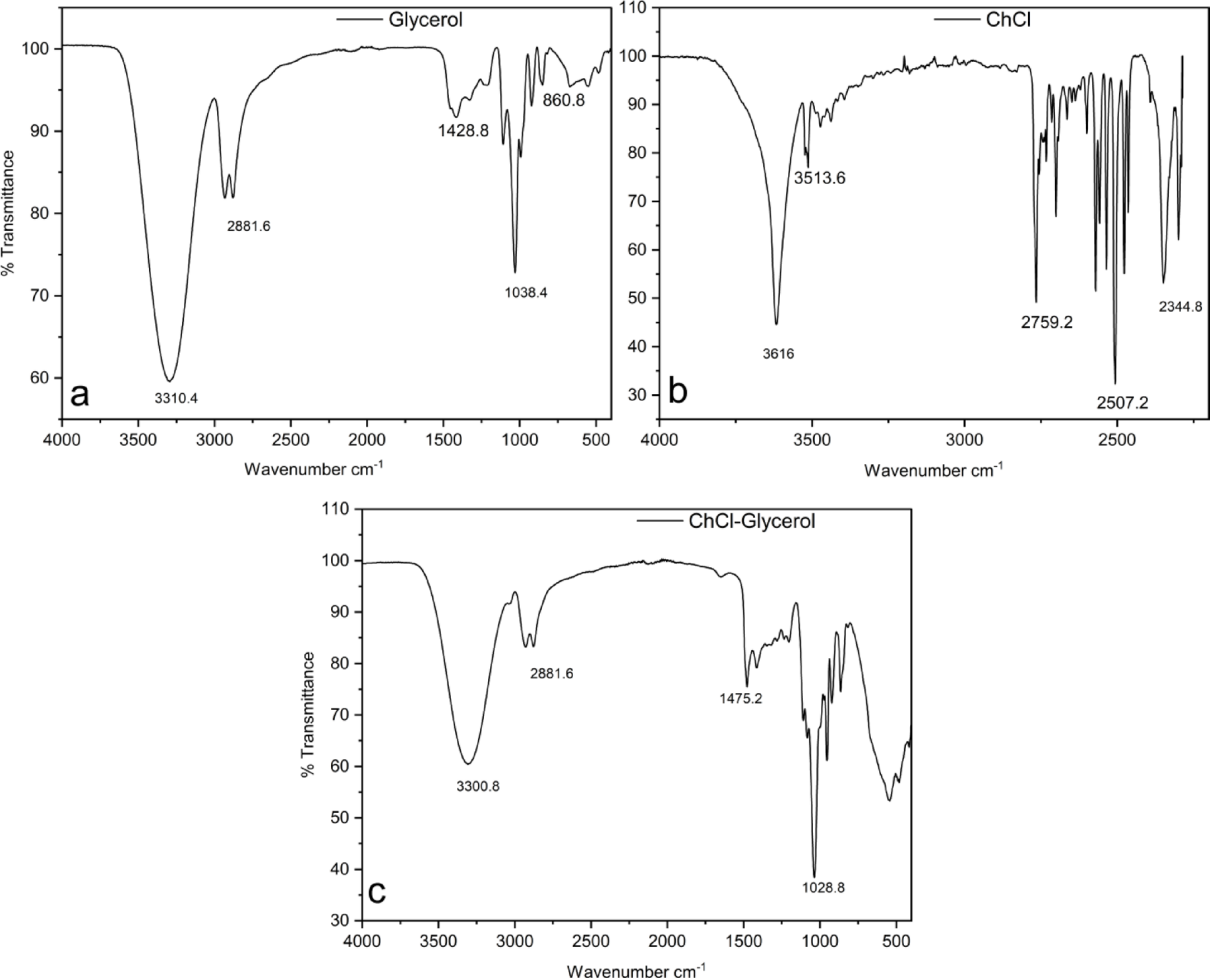
FTIR spectra of DES with individual components.

The FTIR spectra of DES used are presented in Fig. 4, along with individual components (glycerol and choline chloride). For choline chloride (Fig. 4b), the vibrational peaks obtained at around 3616 cm^−1^ and 2759 cm^−1^ indicate the presence of O–H (hydroxyl) and alkyl group (C–H)^39^. In ChCl, the peak at 3513 cm^−1^ can be seen, which indicates the presence of N–H stretching^26^. For glycerol (Fig. 4a), a peak at 3310 cm^−1^ can be assigned to the stretching vibration of the O–H group. Peaks at 3300 in Fig. 4c indicate the O–H group’s stretching vibration after the DES’s formation. Other relevant peaks observed in DES are C–H bonding at 2881 cm^−1^, H–bending at 1475 cm^−1^, and C–C stretching at 1028 cm^−1^. When observing the O–H stretching vibration peak of DES, it is evident that a wavenumber shift towards the HBD (glycerol) side is observed^40^, which indicates that HBD has taken part in the contribution of hydrogen bonding in DES. So, the formation of DES has been confirmed with FTIR.

**Fig. 5:**
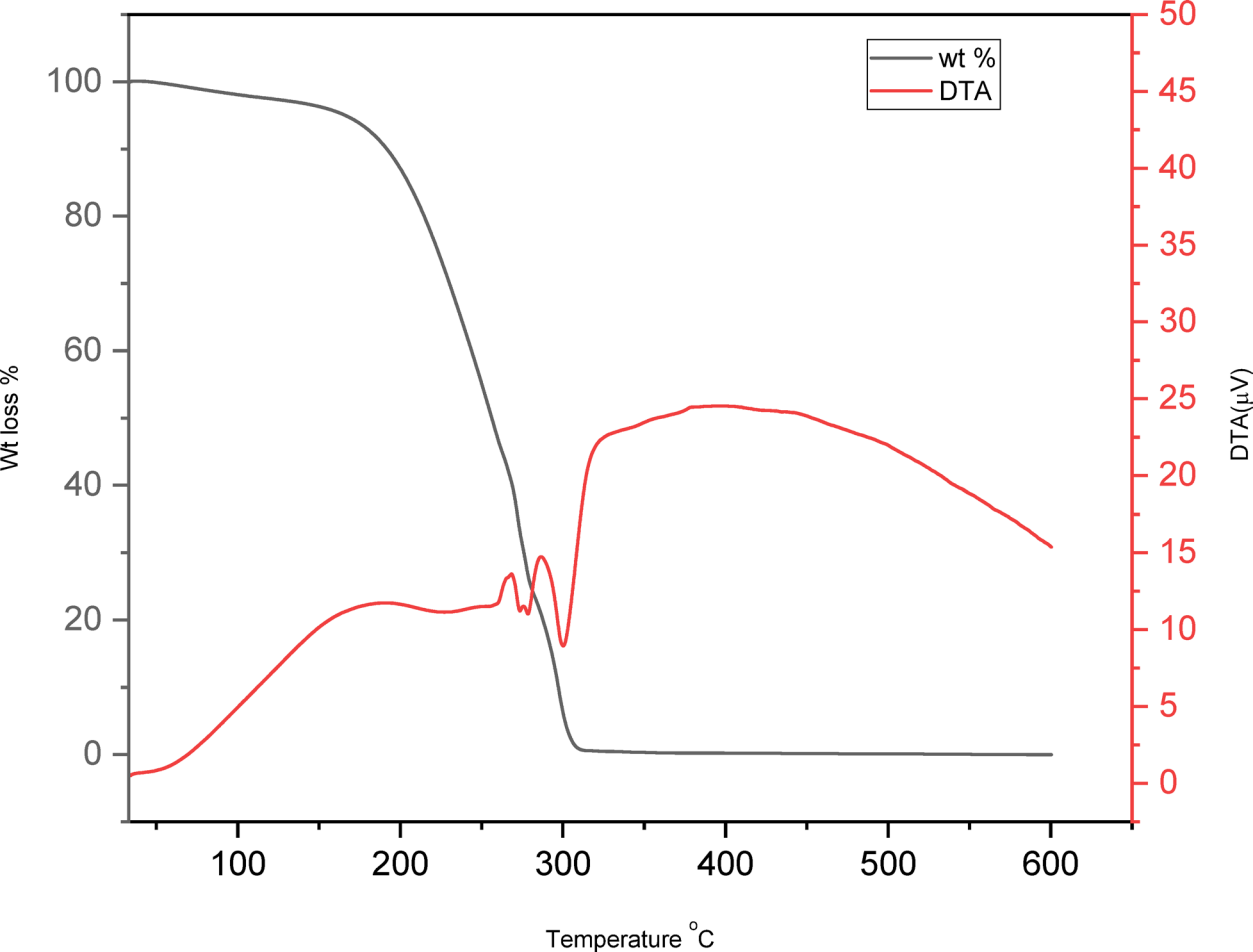
TGA and DTA graphs of DES.

The DES’s thermal stability and onset temperature have been analysed through TG and DTA analysis, as illustrated in Fig. 5. DES experiences a singular mass loss event due to the vaporization of glycerol and the concurrent thermal decomposition of ChCl; these processes transpire simultaneously as the boiling point of glycerol (290 °C) is relatively proximate to the decomposition temperature of ChCl^41^ (260 °C as illustrated in Fig. 5)^42^. The DTA curve indicates that the DES begins to vaporize at approximately 266 °C. The onset decomposition temperature is critical as it establishes the maximum temperature at which deep eutectic solvents can retain their liquid state without decomposition, thereby defining their applicability as solvents. The TG curve indicates an onset temperature of approximately 200 °C, signifying that the DES remains in a liquid state up to that temperature^43^. The decomposition temperature of DES is situated between the decomposition temperatures of its components^44^.

### Characterization of membrane

**Fig. 6:**
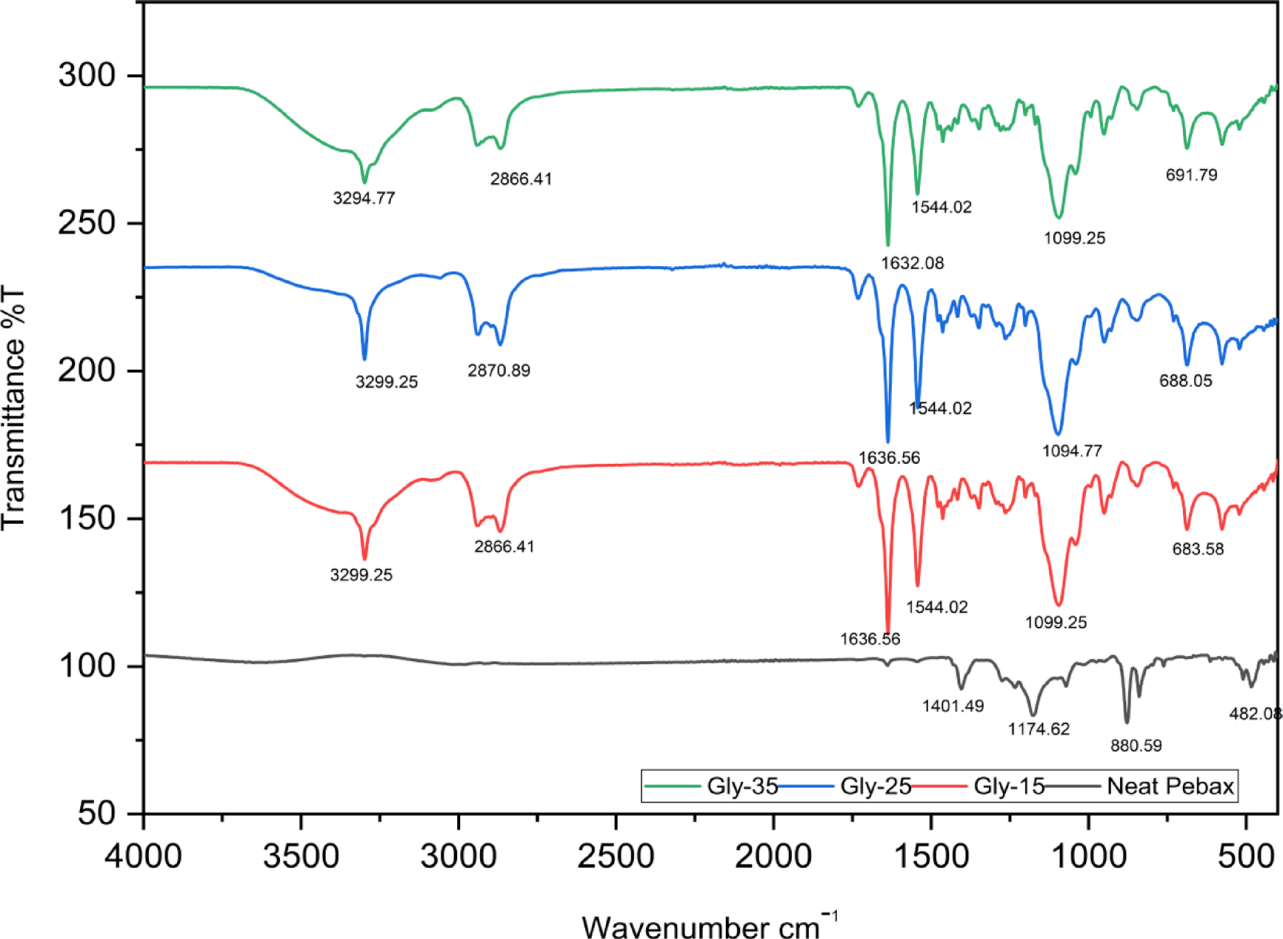
FTIR of Membrane with Neat Pebax.

The FTIR spectroscopy of neat Pebax and DES membranes are shown in Fig. 6. The characteristic peaks of each component are analysed to confirm the fabrication of membranes. The FTIR spectra of neat Pebax-1657 displayed strong bands at 1174 cm^−1^ and 1632 cm^−1^ assigned to the C–O–C stretching vibrations and H–N–C=O vibrations within the polyether segment. Compared with the neat Pebax-1657 membrane, other DES membranes exhibit a strong peak at 3299 cm^−1^, 3299 cm^−1^ and 3294 cm^−1^. This indicated the presence of a hydroxyl group (O–H) in the membrane, which again suggests the presence of DES in membranes^27^. The characteristic peaks of alkyl groups present in ChCl are observed at around 2900 cm^−1^, which again confirms the presence of ChCl in the membrane ^23,25^. The N–H and C–H vibrational bands are observed at 1544 cm^−1^ and 1099 cm^−1^, respectively. Here, with a higher concentration of DES, a wavenumber shift towards the lower side can be observed, indicating a stronger hydrogen bond between HBA and HBD^22,23^. These results reveal that the DES gel membrane has been fabricated well.

**Fig. 7:**
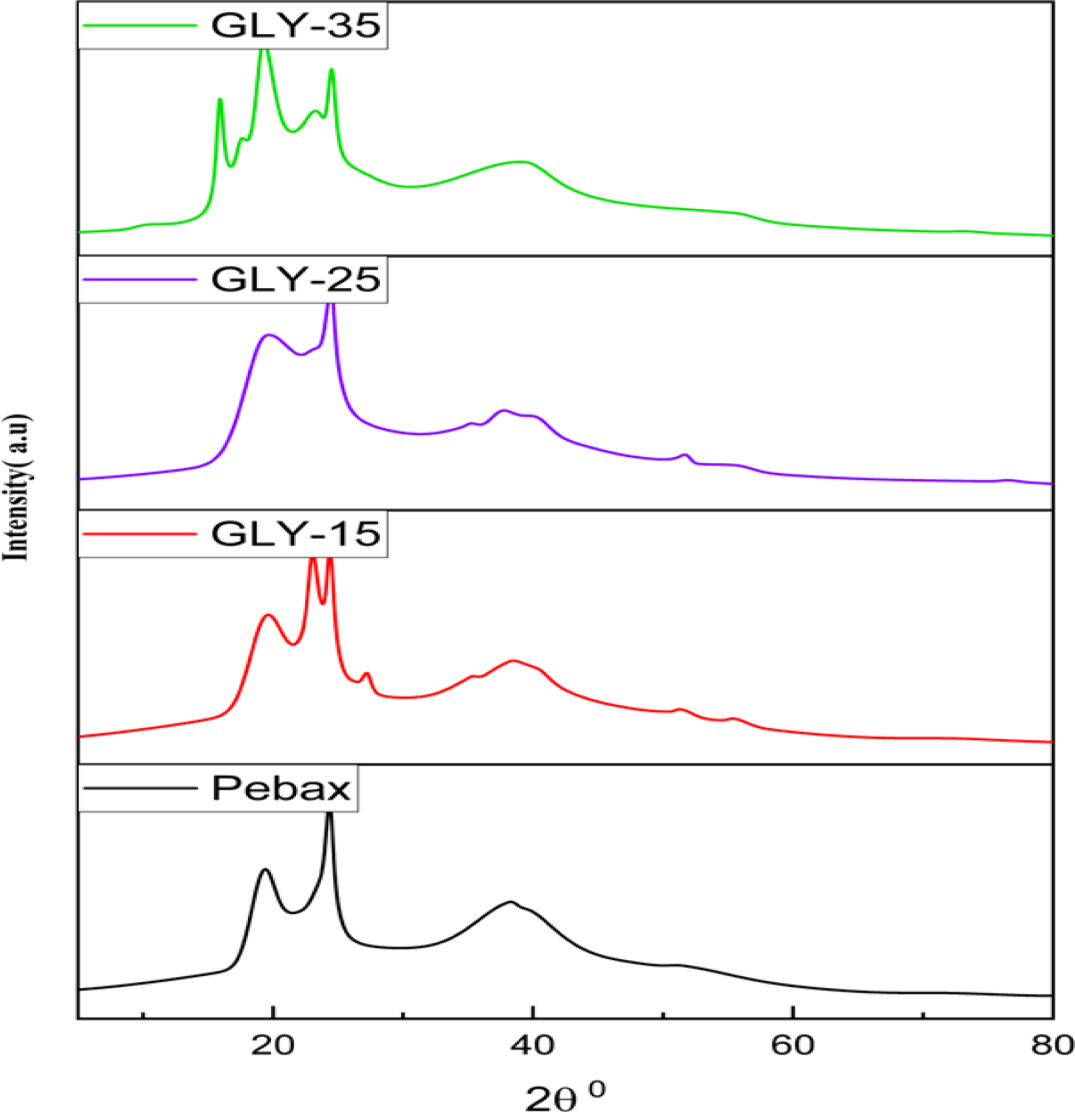
XRD analysis of membranes with Neat Pebax.

In Neat Pebax (Fig. 7), a sharp peak around a 2θ angle of 20° is obtained, which is like the reported values^45^. When it comes to DES gel membranes, the crystallinity of the membrane slowly changes to an amorphous nature, which can be explained by the broadening of peaks at 20°. When the concentration of DES increases, the broadening is also much visible, which indicates that with an increase in addition, the crystalline nature of membranes deteriorates to amorphous; thus, performance is also affected. The presence of choline chloride in the membrane has been confirmed by peaks in the range of 20°–22°^46,47^.

**Fig. 8:**
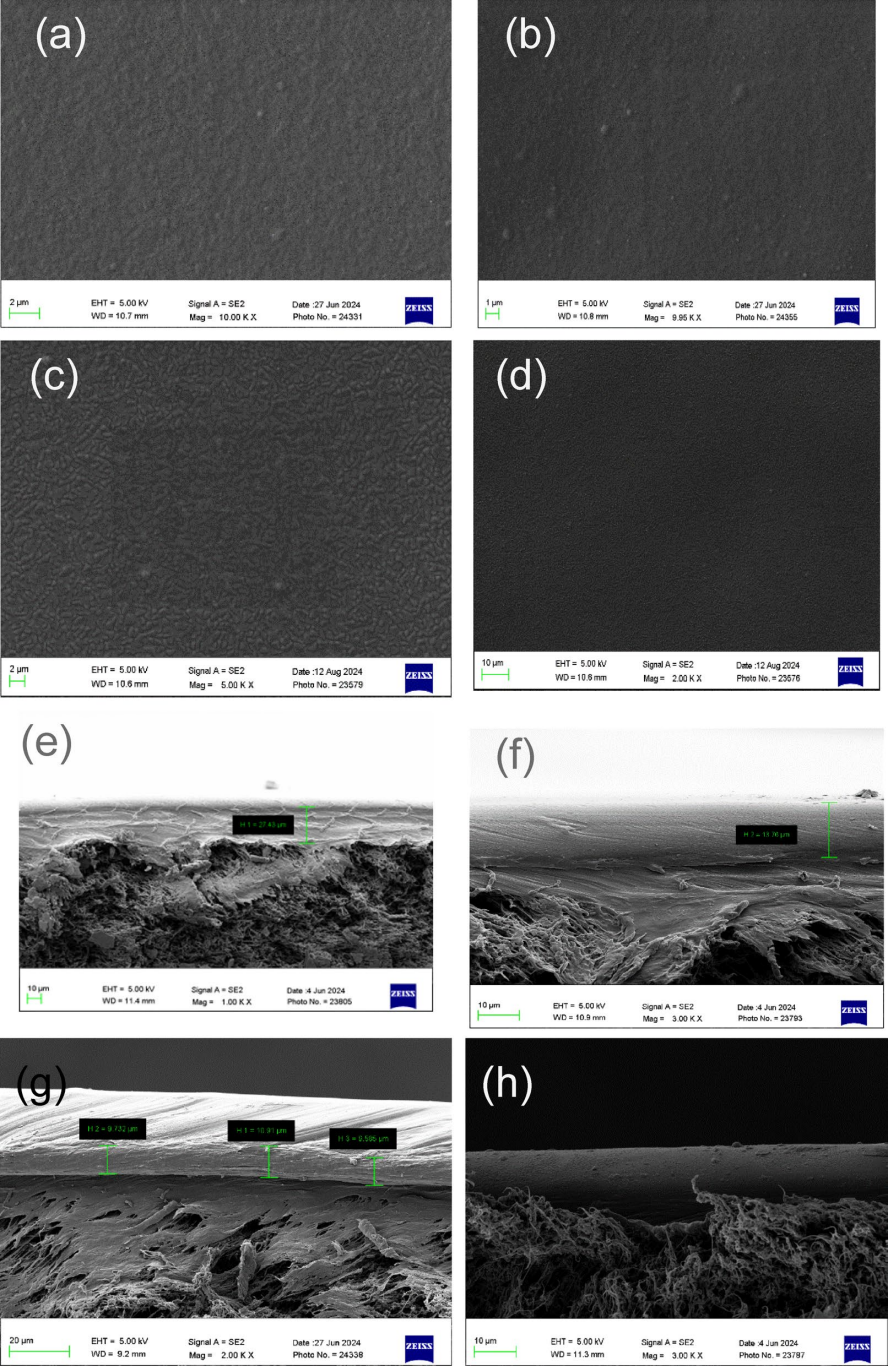
SEM images of (**a**) Neat PEBAX; (**b**) ChCl-glycerol 15%; (**c**) ChCl-glycerol 25%; (**d**) ChCl-glycerol 35%; (**e**–**h**) Cross-section of membranes.

The SEM images of the produced membranes, including pristine Pebax 1657, are given in Fig. 8. Figure 8a illustrates the surface morphology of pristine Pebax, which appears smooth and uniform when contrasted with DES gel membranes. The images indicate that the membrane’s matrix is no longer smooth after adding DES. The surface seems dense, as illustrated in Fig. 8b–d. The increasing quantity of DES renders the membrane rougher than the original Pebax. The addition of DES into a plain polymer membrane disrupts interchain bonding and encourages amorphous segments, potentially disrupting the membrane’s structural uniformity. These hydrogen bonds contribute to preserving the symmetric structure of membranes; their degradation leads to increased amorphousness in the membranes. High concentrations of DES in the membrane show a uniform distribution of DES throughout the membrane surface, which is shown in the SEM images. Figure 8e,f illustrate the cross-section of the DES membrane. The image indicates that the outer fabricated layer of the DES gel membrane is dense, while the lower PVDF support is porous. The outer layer’s thickness is measured at 13.6 µm, which will be utilised for subsequent permeability calculations.

## Membrane performance

**Fig. 9:**
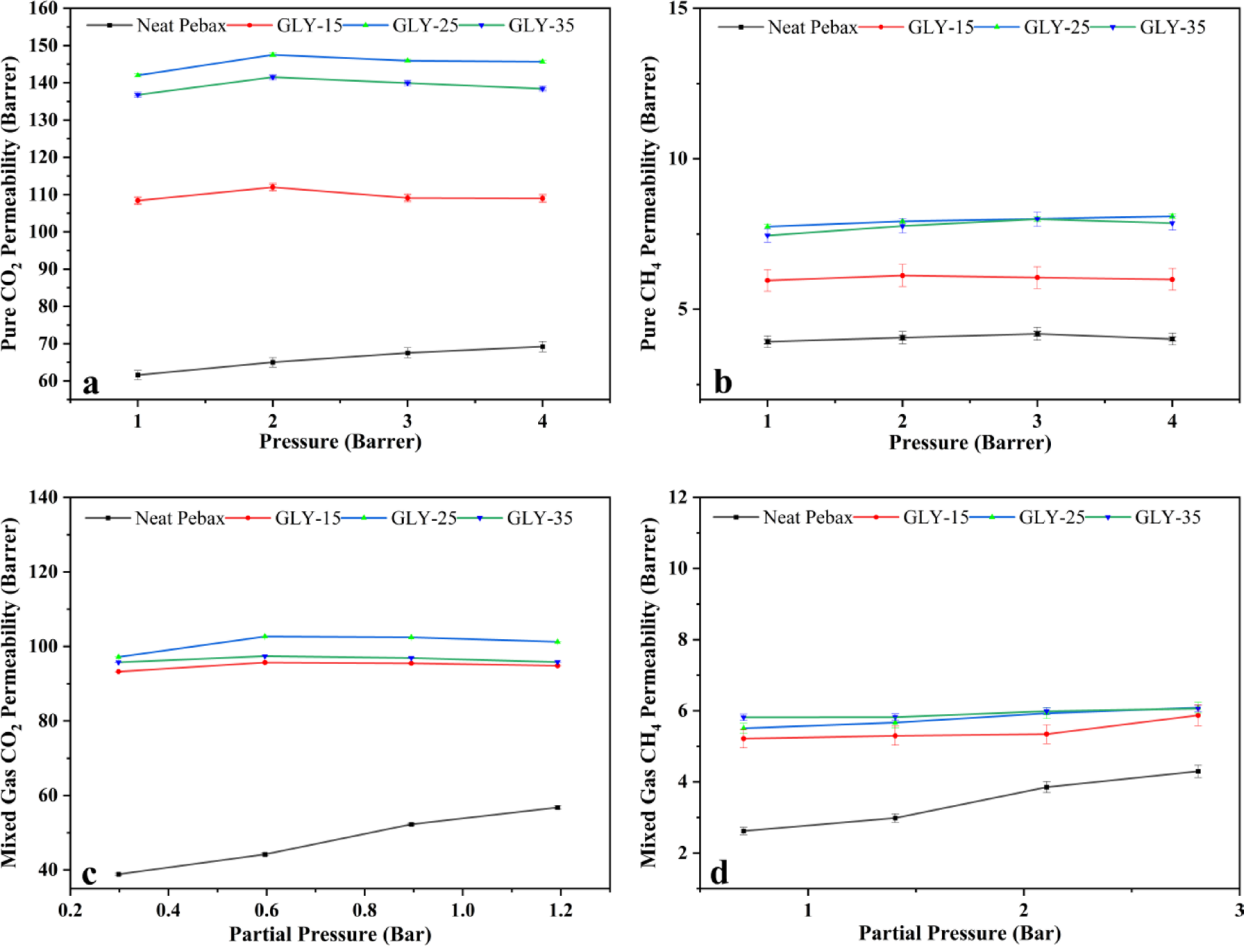
Permeability values of both pure and mixed gases.

**Fig. 10:**
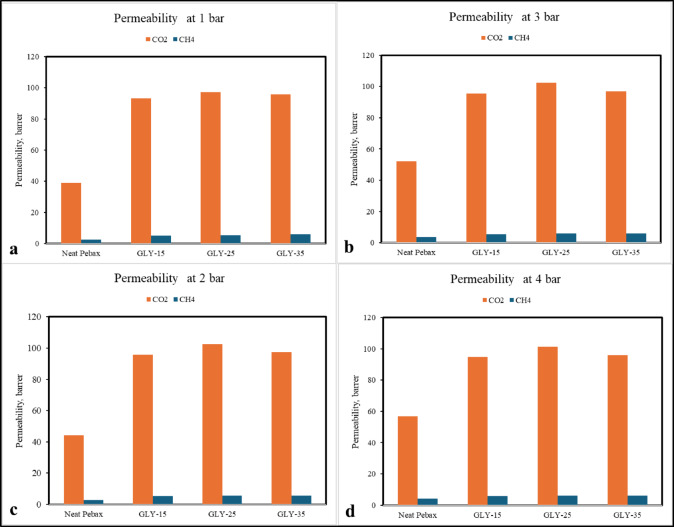
Comparison of permeability values of different DES-loaded membranes with increasing transmembrane pressure.

The manufactured DES gel membranes underwent gas permeation evaluations for both pure and mixed CO_2_ and CH_4_ gases at 35 °C, with feed pressures varying between 1 and 4 bars. All single measurements were conducted in dry conditions. For every membrane, all measurements were conducted a minimum of three times. The permeability and selectivity of fabricated DES ionogel membranes were calculated for both pure gases (CO_2_ and CH_4_) and a mixture of CH_4_/CO_2_ in the ratio 70:30, and the results are plotted in Fig. 9. This figure illustrates the permeability values of both pure and mixed gases through the different concentrated membranes. Here, the GLY-15 membrane indicates it contains ChCl: Glycerol DES in 15% of the weight of the polymer taken; it is the same in the case of GLY-25 and GLY-35. Figure 10 explains the comparison of the permeabilities of mixed gases under different pressures. All three membranes with Neat Pebax are tested for permeability measurement. The solution diffusion mechanism affects gas permeation through the DES ion membrane. As observed, gas transport happens in three steps. In the first step, gas gets absorbed on the membrane surface, which has solvents that have an affinity for a particular gas. In the second step, it gets diffused. It gets desorbed through the membrane to the other side of the membrane and, in the third step, on the permeate side at low pressure^48^.

From Fig. 10, it is evident that the permeability values of pure CO_2_ are way higher than those of pure CH_4_. The highest pure CO_2_ gas permeability value is obtained for GLY-25, which is around 147.5 Barrer, whereas the CO_2_ Permeability of Neat Pebax is in the range of 40–60 Barrer.

Tables 7 and 8 show the permeability and selectivity values of both pure gas and mixed gases calculated for different membranes. The permeability values are calculated using the thickness and area of the cut membrane, which is kept in the membrane module. The selective layer thickness of the dense Pebax layer is calculated from Scanning Electron Microscopy (SEM) of the membrane cross-section. For example, the SEM cross-section image of the GLY-15 membrane is shown below, which indicates a thickness of 13.76 µm (Fig. S2). Similar way thickness of all the membranes is calculated and Table 9 below shows the other values.

**Table 7:** Permeability of pure CO_2_ and pure CH_4_.

Pressure (bar)	Permeability (Barrer), Pure CO_2_				Permeability (Barrer), Pure CH_4_				Selectivity			
	Neat Pebax	GLY-15	GLY-25	GLY-35	Neat Pebax	GLY-15	GLY-25	GLY-35	Neat Pebax	GLY-15	GLY-25	GLY-35
1	61.58	108.39	142.02	136.79	3.92	5.96	7.75	7.45	15.69	18.19	18.33	18.35
2	65.01	111.97	147.5	141.55	4.06	6.12	7.93	7.77	16.03	18.29	18.61	18.22
3	67.56	109.13	145.94	139.96	4.18	6.05	8.01	8	16.13	18.03	18.23	17.5
4	69.21	108.97	145.68	138.44	4.01	6.79	8.09	7.86	17.24	18.1	18	17.61

**Table 8:** Mixed gas permeability of CO_2_ and CH_4_.

Press (bar)	Permeability (Barrer), Mixed CO_2_				Press (bar)	Permeability (Barrer), Mixed CH_4_				Press (bar)	Selectivity			
	Neat Pebax	GLY-15	GLY-25	GLY-35		Neat Pebax	GLY-15	GLY-25	GLY-35		Neat Pebax	GLY-15	GLY-25	GLY-35
0.3	38.85	93.26	97.17	95.73	0.7	2.62	5.22	5.51	5.82	0.3	14.82	17.87	17.64	16.45
0.6	44.20	95.73	102.68	97.37	1.4	2.98	5.30	5.67	5.82	0.6	14.81	18.06	18.11	16.72
0.89	52.25	95.49	102.47	96.88	2.11	3.85	5.34	5.93	5.99	0.89	13.57	17.87	17.28	16.18
1.19	56.82	94.82	101.26	95.82	2.81	4.30	5.87	6.09	6.06	1.19	13.22	16.14	16.63	15.80

**Table 9:** Thickness and cross-sectional area of DES-Gel membranes.

Membrane	Thickness (µm)	Area of cut membrane (cm^2^)
GLY-15	13.76	4.90625
GLY-25	10.07	4.90625
GLY-35	11.07	4.90625

### Effect of transmembrane pressure

The permeability of each membrane changes with an increase in transmembrane pressure from 1 to 4 bar, as shown in Fig. 9. With an increase in partial pressure, the permeability increases first; after that, an increase in pressure results in a decrease in permeability^49^. The same trend is observed in all the membranes. Lian et al.^24^ in their CO_2_ separation study, have obtained similar results, indicating that the transfer process of CO_2_ through the membrane is sensitive to pressure changes. The initial increase in permeability was due to the higher solubility of CO_2_ in DES; however, a further increase in pressure might have caused a pressure-induced compression of the network structure of the membrane. The GLY-35 membrane exhibits lower permeability, where the highest loading of DES is present, compared to GLY-15 and GLY-25.

The permeability of gases through the gel membrane can be explained in different ways. First, by using hole theory, in theory, free spaces exist between the DES’s ionic constituents that allow ionic motion in the DESs^22,23^. Ion-HBD interactions control these holes. Secondly, using the Camper model suggests that CO_2_ permeability is significantly higher for liquids with lower molar volume. Many DES have low molar volume compared to Ionic Liquids, thus exhibiting high CO_2_ solubility^50^. The solubility of CO_2_ is also affected by the basicity of the solvent. As ChCl is basic in nature, increasing its amount directly increases its permeability value. So, in both pure gas and mixed gas studies, it is observed that the permeability of GLY-25 is higher than that of GLY-15 and GLY-35. Though we were expecting an increase in permeability with an increase in DES concentrations, practically, a decline in the permeability value was observed for higher concentrations. This can be explained in the following way: at higher DES concentrations (> 25%), there could be phase separation or the formation of microdomains within the membrane. This means that the DES molecules may start to cluster together or even form small liquid phases, disrupting the uniformity of the membrane. These clusters can create non-uniform channels or blockages that hinder the movement of CO_2_, thus reducing permeability. An additional possibility might be that the plasticization of membrane DES, being a solvent, can act as a plasticizer at higher concentrations, disrupting the membrane’s structure; also, increasing the concentration of DES might change the membrane structure to be more amorphous. These lead to membrane integrity changes, causing it to lose its ideal gas transport properties. While moderate amounts of DES could enhance flexibility and CO_2_ transport, excessive DES might cause the membrane to become too fluid or unstable, altering the balance between gas solubility and diffusivity. This destabilization can lead to a loss of selective gas transport pathways, negatively affecting CO_2_ permeability. DFT study also supports the above statement as the stabilization energies of DES with CO_2_ are almost 8 times higher than CH_4_. So, the absorption rate is much higher than the desorption rate, thus reducing the permeability at higher pressure and increasing DES concentrations.

**Fig. 11:**
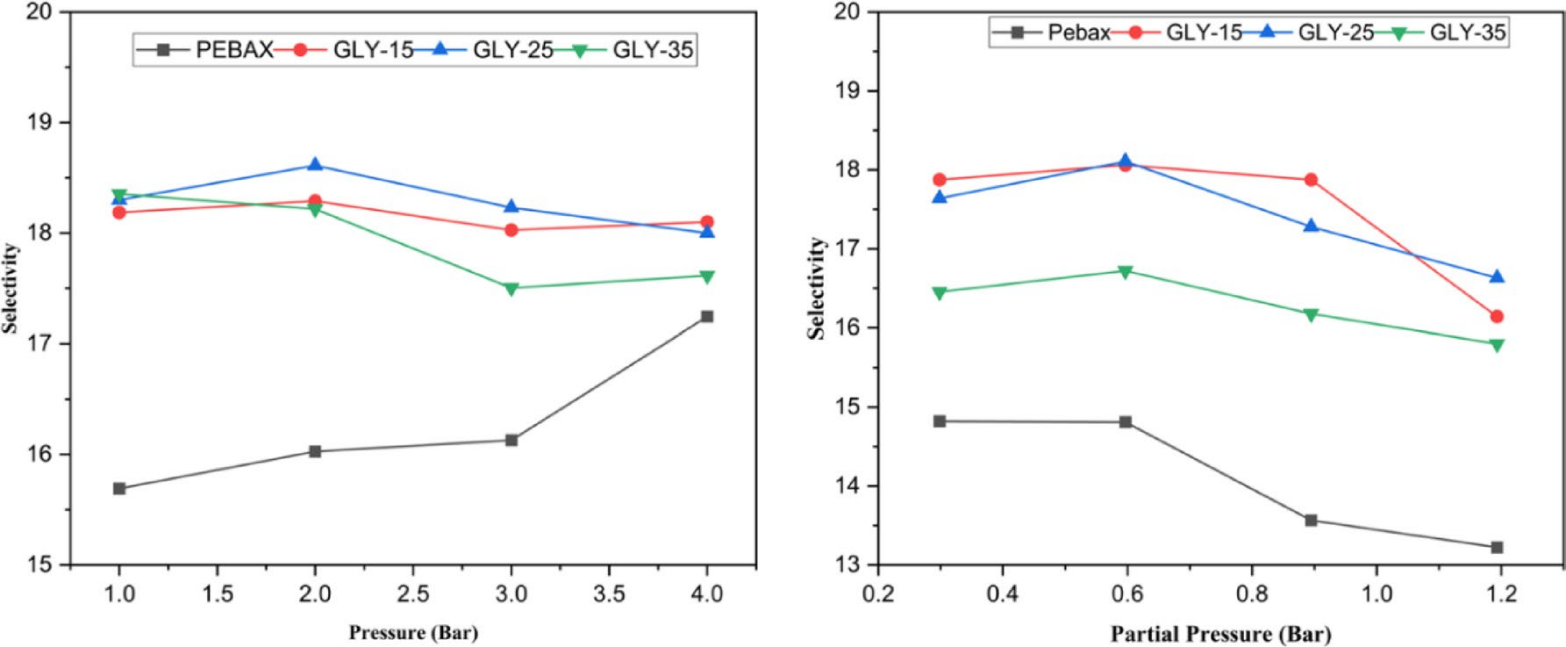
Comparison of selectivity values of pure and mixed gases for different DES-loaded membranes with increasing transmembrane pressure.

**Fig. 12:**
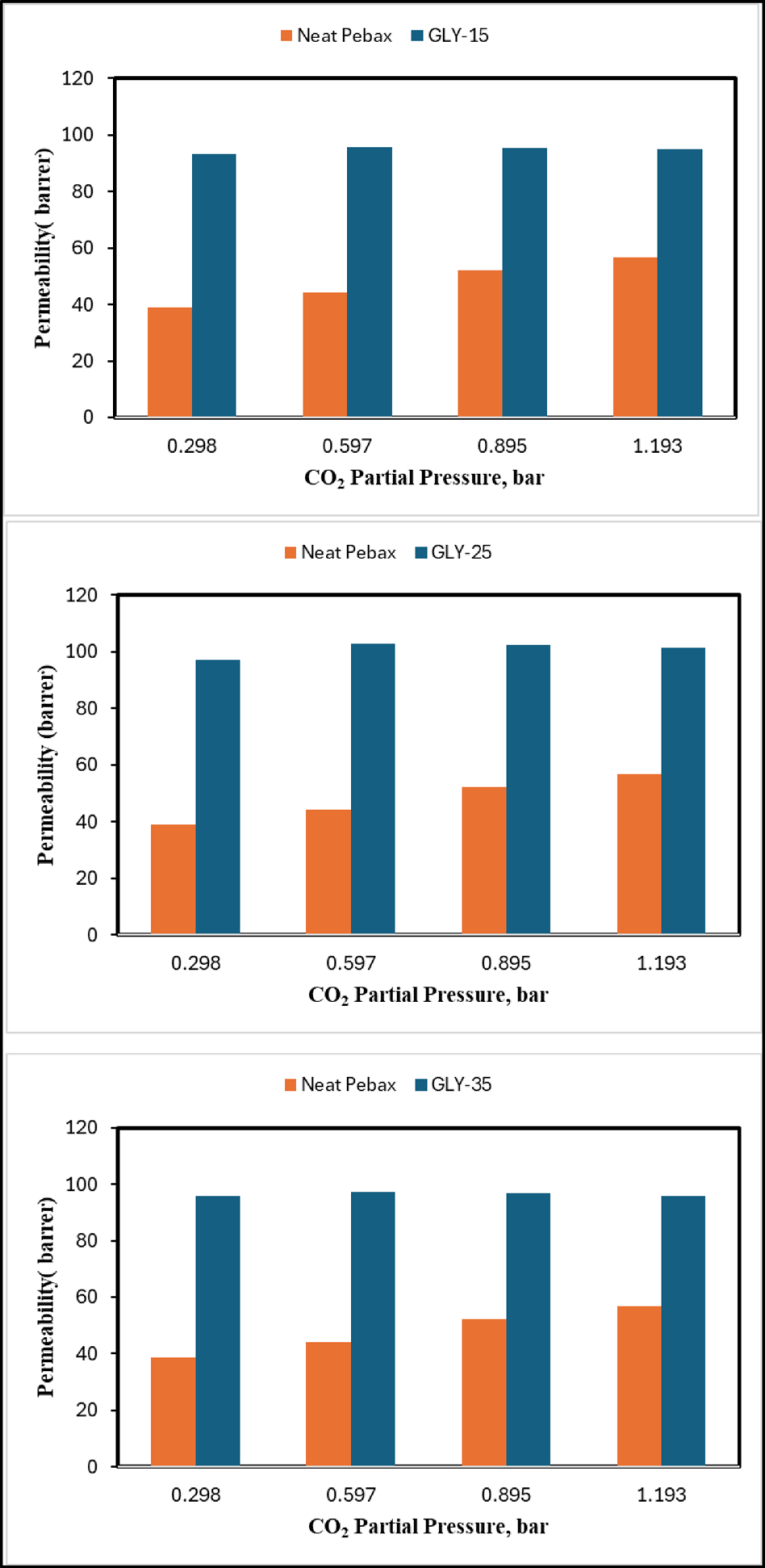
Permeability enhancement of gel membranes over Neat Pebax with increasing transmembrane pressure.

A plot of selectivity values of pure and mixed gases is shown in Fig. 11. Initially, the selectivity for CO_2_ relative to CH_4_ generally enhances with rising pressure. The elevated pressure amplifies the concentration gradient across the membrane, favouring the more soluble CO_2_ in comparison to CH_4_. Research indicates that membranes containing DES additives demonstrate elevated selectivity ratios owing to their capacity to preferentially absorb CO_2_. The enhancement of permeabilities of Neat Pebax over DES gel membranes at different pressures is shown in Fig. 12. It can be observed that at lower pressures, GLY-25 shows an almost 130% increase in permeability compared to Neat Pebax. At the same time, GLY-15 and GLY-35 show permeability enhancement by nearly 125% and 100%, respectively. Although the DES gel membranes exhibit excellent permeability, the selectivity of the membranes is comparatively lower.

At elevated pressures, selectivity may diminish. This can be explained as the membrane structure’s rigidification under high pressures can reduce interactions between the polymer matrix and gas molecules, adversely affecting selectivity^38^. As pressure escalates, both gases may undergo augmented transport rates across the membrane, resulting in a diminished efficiency of the separation process due to the convergence of their permeabilities^20^. DES components are frequently physically encapsulated within the polymer matrix through hydrogen bonding rather than through chemical bonding. For instance, in PVA-based DES membranes, FTIR analysis verified that DES is physically absorbed, resulting in potential leaching risks under stress^51^. Elevated pressures can diminish DES viscosity, compromising its retention within the membrane. In ChCl-1,2-propanediol DES membranes, CO₂ permeability decreased by 20–30% under increased pressures, indicating potential DES displacement or leaching^26^. Tetrabutylammonium chloride-based deep eutectic solvents exhibited diminished leaching in CO_2_/CH_4_ separation owing to decreased water affinity. Membranes containing 15–20 wt% DES loading optimize performance and stability while reducing leaching. DES gel membranes are susceptible to leaching under high pressure if inadequately stabilized. Crosslinking, the selection of hydrophobic DES, and optimized loading can alleviate this risk, guaranteeing stable performance in applications such as CO_2_/CH_4_ separation. Elevated pressure can destabilize DES gel membranes by causing structural alterations and facilitating the leaching of DES components, especially in non-crosslinked or hydrophilic systems. Elevated pressure diminishes the viscosity of DES, compromising its physical entrapment within the polymer matrix. For instance, CO_2_ permeability in ChCl-1,2-propanediol deep eutectic solvent membranes decreased by 20–30% under increased pressures, suggesting DES displacement or leaching.

The gas permeability values obtained from this work are compared with other literature in Table 10, which uses supported DES membranes. Among all the published research on DES SLMs, this work introduces DES-gel membranes and exhibits a very high permeability value compared to the existing literature. Therefore, this membrane casting technique can be a promising approach to achieving desirable permeability. The casting technique requires a minimal amount of DES; hence, solvent wastage is eliminated. Nowosielski et al.^26^ obtained higher permeability in their work using the wet impregnation method. A larger amount of solvent is required for the fabrication process, which can be considered a limitation of their work.

**Table 10:** Comparison with other DES-membranes.

S. No	DES		Polymer	CO_2_ permeability	References
	HBA	HBD			
1	Choline chloride	MEA, DEA, TEA	PVDF	25–28	^23^
2	Thymol	Coumarin	Hydrophobic PVDF	75	^24^
3	Betaine	Fatty acids	Microporous PVDF	31.23–35.67	^25^
4	Choline chloride, acetyl choline chloride	1,2 propanediol	Polypropylene	86–152	^26^
5	L-arginine	Ethylene glycol	Pebax 1657	65	^52^
6	Choline chloride	Malic, oxalic, Tartaric	Microporous PVDF	30–37	^53^
7	Choline chloride	Polyacrylic acid, polyacrylamide	PVDF	20–27	^21^
8	Choline chloride	Glycerol	Pebax1657	147.49	This work

**Fig. 13:**
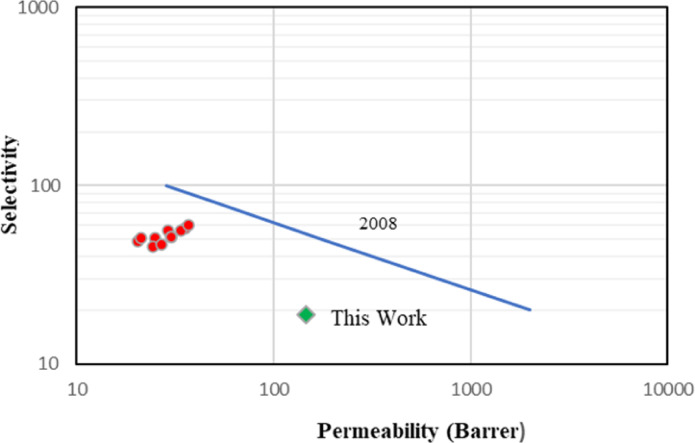
Robeson plot to analyse the permeability and selectivity of the current DES gel membrane.

The Robeson plot has been plotted in Fig. 13 to analyse the permeability and selectivity of the current DES gel membrane, and it is found that the membrane proves to be excellent in permeability; however, selectivity must be compromised^54,55^. Other relevant work on DES membranes is plotted in red dots, specifically for CO_2_/CH_4_ separation. The current membrane has been plotted in green dots.

## Conclusions

This study employs a novel technique for membrane fabrication and explores a new composition of Pebax 1657 combined with DES for separating CO_2_ from a CO_2_/CH_4_ mixture. The composition demonstrates a strong affinity for CO_2_. Various concentrations of DES have been incorporated into the polymer for membrane fabrication. The influence of transmembrane pressure and DES concentration on the permeability values of pure and mixed gases has been analysed. The permeabilities of both pure and mixed gases initially increase and subsequently decrease with pressure. This is attributable to the deformation of the membrane structure under elevated pressure. The membrane demonstrates the highest mixed gas permeability of 102.68 Barrer and a pure gas CO_2_ permeability of 147.5 Barrer at a concentration of 25%. Results of neat Pebax membrane with DES-gel membrane have been compared at different pressures, and a 75% increase in CO_2_ permeability was found. Further research in this field can be extended to enhance the selectivity of the membrane. The elevated permeability values and cost-effectiveness make them viable candidates for DES gel membranes.

## Supplementary Information

Below is the link to the electronic supplementary material.

Supplementary Material 1

## Data Availability

The datasets used and analysed during the current study are available from the main corresponding author (Dr. Swapnil Dharaskar) on reasonable request.
