# Supplementary material for: Experimental and predictive analysis of deep eutectic solvent gel membranes for efficient CO2 separation
Source: Sci Rep. 2025 Aug 13;15:29677. doi: 10.1038/s41598-025-14520-z (PMC12350861; doi:10.1038/s41598-025-14520-z)
Supplement: Supplementary file 1 — Supplementary Material 1 [file 41598_2025_14520_MOESM1_ESM.docx]

**Scientific Reports**

**Experimental and Predictive Analysis of Deep Eutectic Solvent Gel Membranes for Efficient CO₂ Separation**

*Remya Ranjith^1^, Bharti Saini^1^, Swapnil Dharaskar^1*^, Tushar Patil^1^, Grishma Pindolia^2^, Satyam Shinde^2^, Rama Rao Karri^3^**

^1^Department of Chemical Engineering, School of Energy Technology, Pandit Deendayal Energy University, Gandhinagar, Gujarat-382426

^2^Department of Physics, School of Energy Technology, Pandit Deendayal Energy University, Gandhinagar, Gujarat-382426

^3^Chemical and Energy Engineering, Faculty of Engineering, Universiti Teknologi Brunei, Bandar Seri Begawan, BE1410, Brunei Darussalam.

**Corresponding Author(s)^*^**: swapnil.dharaskar@sot.pdpu.ac.in, swapnildharaskar11@gmail.com (SD); kramarao.iitd@gmail.com (RRK)

**Supplementary Information**


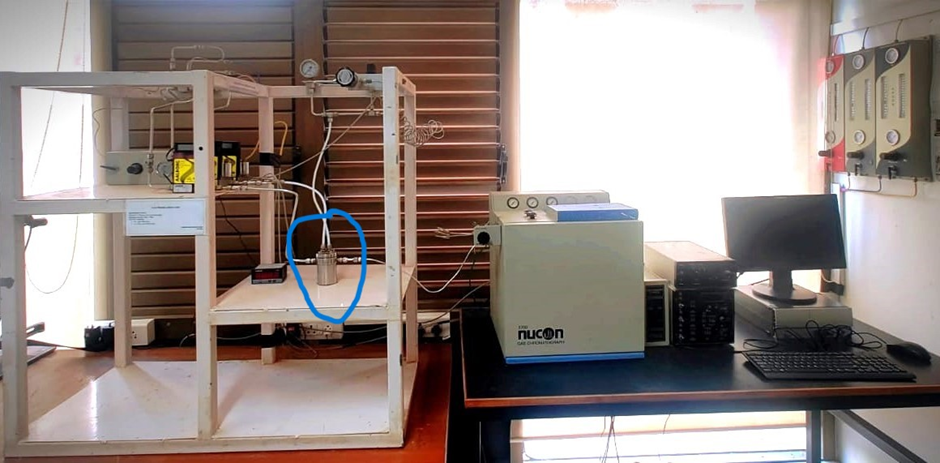


**Figure S1:** Actual image of Experimental Setup


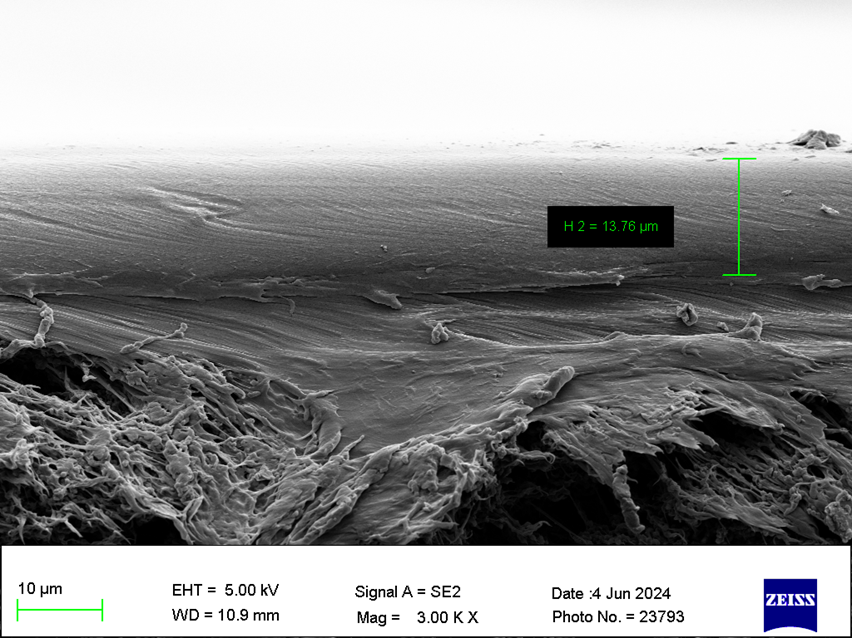


**Figure S2:** Cross-Sectional image of GLY-15 Membrane

- 1. ***Computational Methodology (Supplementary information)***

The interaction energies (*E_int_*) of the DES-CO_2_ and DES-CH_4_ complexes have been calculated using Equation 1.

$\boldsymbol{E}_{\boldsymbol{int}}\boldsymbol{=}\boldsymbol{E}_{\boldsymbol{com}}\boldsymbol{-[EDES+}\boldsymbol{E}_{\boldsymbol{gas}}\mathbf{]}$ **(1)**

Here, $E_{DES}$, $E_{gas}$, and $E_{com}$ represents optimised structure energies of DES, gas molecules, and their corresponding complexes. Enthalpy change ($\Delta H)$ and Gibbs free energy ($\Delta G$) for the adsorption process have been calculated from the frequency analysis at room temperature.

$\boldsymbol{\Delta H}\boldsymbol{=}\boldsymbol{H}_{\boldsymbol{com}}\boldsymbol{-[}\boldsymbol{H}_{\boldsymbol{DES}}\boldsymbol{+}\boldsymbol{H}_{\boldsymbol{gas}}\mathbf{]}$ **(2)**

$\boldsymbol{\Delta G}\boldsymbol{=}\boldsymbol{G}_{\boldsymbol{com}}\boldsymbol{-[}\boldsymbol{G}_{\boldsymbol{DES}}\boldsymbol{+}\boldsymbol{G}_{\boldsymbol{gas}}\mathbf{]}$ **(3)**

The gas adsorption selectivity ($S_{CO2/CH4}$) of DES is defined as follows:

$\boldsymbol{S}_{\boldsymbol{CO}\boldsymbol{2/CH}\boldsymbol{4}}\boldsymbol{=}\boldsymbol{\Delta G}_{\boldsymbol{CO}\boldsymbol{2}}\boldsymbol{-}\boldsymbol{\Delta G}_{\boldsymbol{CH}\boldsymbol{4}}$ **(4)**

The Electrostatic Potentials (ESP) and Frontier molecular orbitals (FMO) of DES, gases, and their complexes were analysed and visualised by Gauss View 5. The Reduced Density Gradient (RDG) analysis and Natural Bond Orbital (NBO) analysis have also been carried out to study the interaction, especially called non-covalent interactions (NCI). This NCI causes stability to complexes. Multiwfn, a software package, has been utilised to generate the NCI-RDG plots, and the Visual Molecular Dynamics (VMD) software package has been used to create the 3D RDG plots.

3.3

- 1. ***Density Functional Theory (DFT)***

The optimised molecular geometries of DES and gas molecules are shown in **Figure S3**. The optimised structures with significant donor-acceptor interactions are shown in **Figure S4**. The distance between O39 (of CO_2_ molecule) and H21 (of DES molecule) in the DES-CO_2_ complex is 3.937Å, and the distance between O38 (of CO_2_ molecule) and H30 (of DES molecule) is 2.664Å. The distance between H41 (CH_4_ molecule) and O24 (DES molecule) in the DES-CH_4_ complex is 2.575Å. The linear angle of the CO_2_ molecule changes to 176.890° after adsorption by DES to form the DES-CO_2_ complex. A significant change in the molecular structure of CH_4_ does not accompany the adsorption of CH_4_ by DES. The ESP maps of the complexes are shown in **Figure S5**. The positive and negative potentials are indicated by the blue and red regions, respectively, in the maps. This makes the ESP maps helpful in constructing the initial structures of complexes because nucleophilic and electrophilic regions of the isolated molecules interact to form a stable structure. The FMO (Fragment Molecular Orbit), which includes the lowest unoccupied molecular orbital (LUMO) and highest occupied molecular orbital (HOMO), highlights the reactivity of the molecules. The FMO of DES and the complexes and their energy gaps (*E_g_*) are displayed in **Figure S6**. There is no contribution to the FMO of the DES-gas complexes from the gas molecule side. The absence of a remarkable change in E_g_ indicates weak interactions between DES and gas molecules in both complexes. A deeper insight into these weak interactions will be obtained from the NBO and NCI-RDG analysis.

The values of E_int_ (kJ/mol) for DES-CO_2_ and DES-CH_4_ are observed to be -8.546 and -1.449, respectively. The negative values of the E_int_ of complexes are indicative of their stability. The E_int_ of DES with CO_2_ is approximately 6 times that with CH_4_. Hence, the DES-CO_2_ complex is much more stable than the DES2-CH_4_ complex. The adsorption process of both the gas on DES is exothermic, as shown by the negative value of ${\Delta H}$(kJ/mol) for DES-CO_2_ (-8.835) and DES-CH_4_ (-0.433) complex. The negative value of $S_{CO2/CH4}$ (-5.036 kJ/mol) suggests that CO_2_ has more affinity towards DES than CH_4_. Hence, CO_2_ will be adsorbed more effectively by DES than CH_4_.


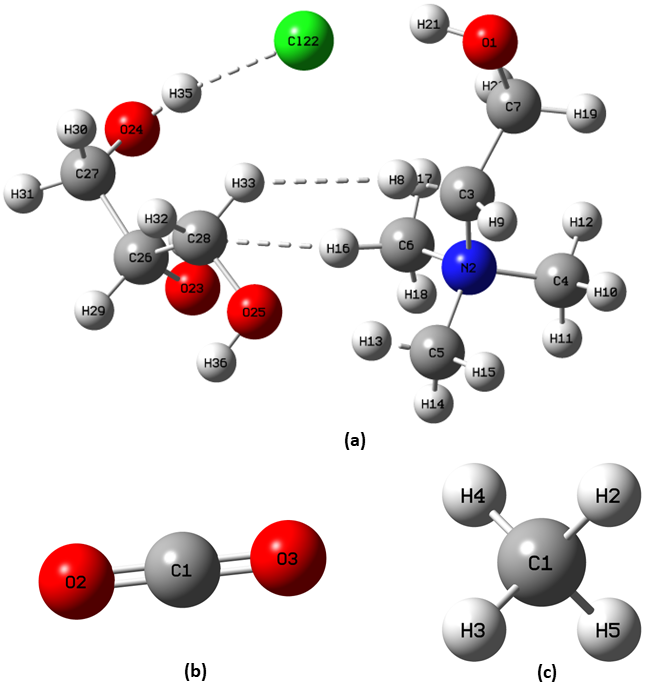


**Figure S3: Optimised geometries of (a) DES; (b) CO2 (c) CH4**

***
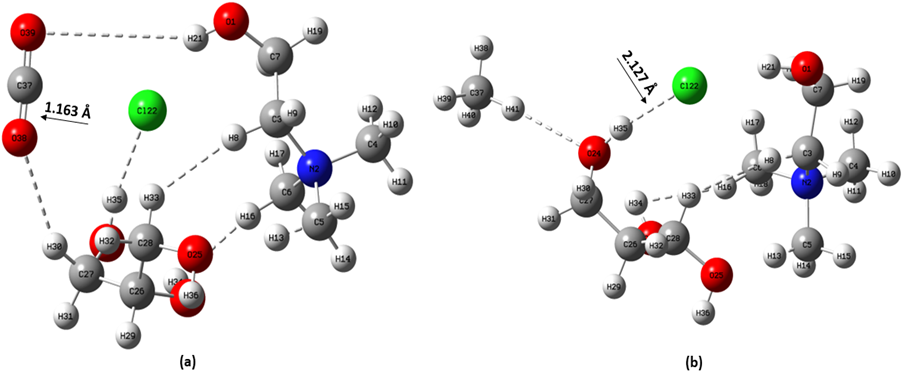
***

**Figure S4: Optimised Structures with significant donor-acceptor interaction of (a) DES-CO_2_; (b) DES-CH_4_ Complexes**


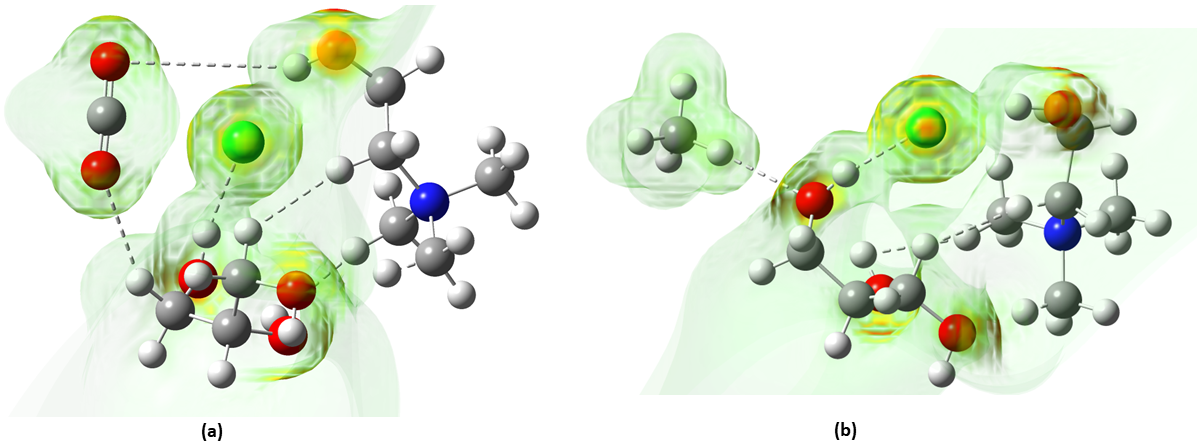


**Figure S5: ESP Mapping of (a) DES-CO_2_; (b) DES-CH_4_ Complexes**


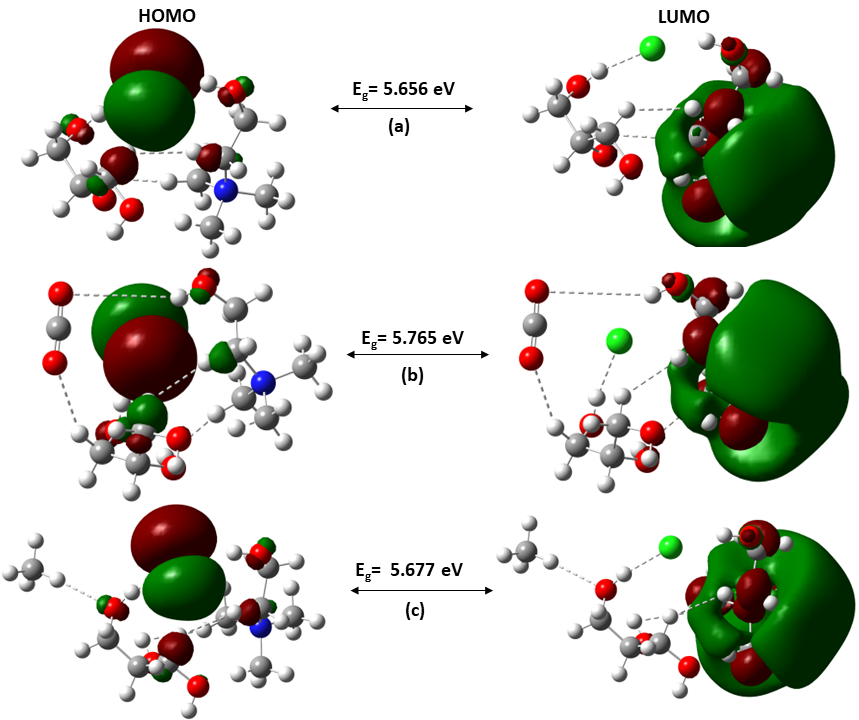


**Figure S6 FMO of (a) DES; (b) DES-CO_2_; (c) DES-CH_4_**

:

The stabilization energy (E_2_) obtained from the NBO analysis is used to study the charge transfer interactions. E_2_ represents the energy required for electron delocalization between donor and acceptor, and hence, it is helpful to characterize the interactions between DES and gas molecules. In the case of the DES-CO_2_ complex, the highest interaction is between the lone pair of oxygen atoms (O38) and σ* (C37-O39) of the CO_2_ molecule. The most significant interaction in the DES-CH_4_ complex is found between the lone pair of chlorine (Cl22) and σ* (O24-H35) of the DES molecule. DES-CO_2_ shows a higher value of E_2_ than DES-CH_4_. This indicates the presence of stronger interactions between DES and CO_2_ than between DES and CH_4_. The weak hydrogen bonds (WHB) and Van der Waals (VDW) interactions are key contributors to the stability of supramolecular complexes. The RDG confirms their presence in both complexes’ vs (sign λ₂)ρ plots shown in **Figure S7**. The second eigenvalue of the Hessian matrix (λ₂) is negative for attractive interactions and positive for repulsive interactions. The low values of RDG are indicative of some weak chemical interactions. The value of interactions is determined by the density of the electron (ρ). The VDW interactions are characterised by a low value of RDG (<0.5) and a value of (sign λ₂)ρ between -0.01 a.u. and 0 a.u. The WHB is characterised by the value of (sign λ₂)ρ between 0.02 a.u. and 0.05 a.u. From these plots, we can confirm that stronger NCI stabilizes the DES-CO2 complex. **Figure S8** shows the regions of these NCI for both complexes through the RDG iso-surfaces. The bluish-green disks demonstrate the WHB, and the green-coloured patches show the VDW interactions.


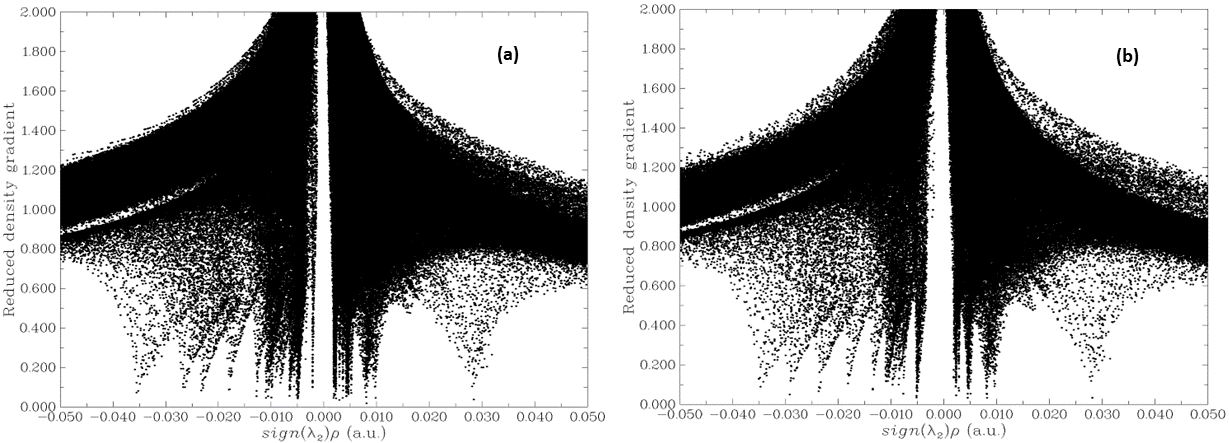


**Figure S7: RDG vs (sign λ₂)ρ plots for (a) DES-CO_2_ and (b) DES-CH_4_ complex**


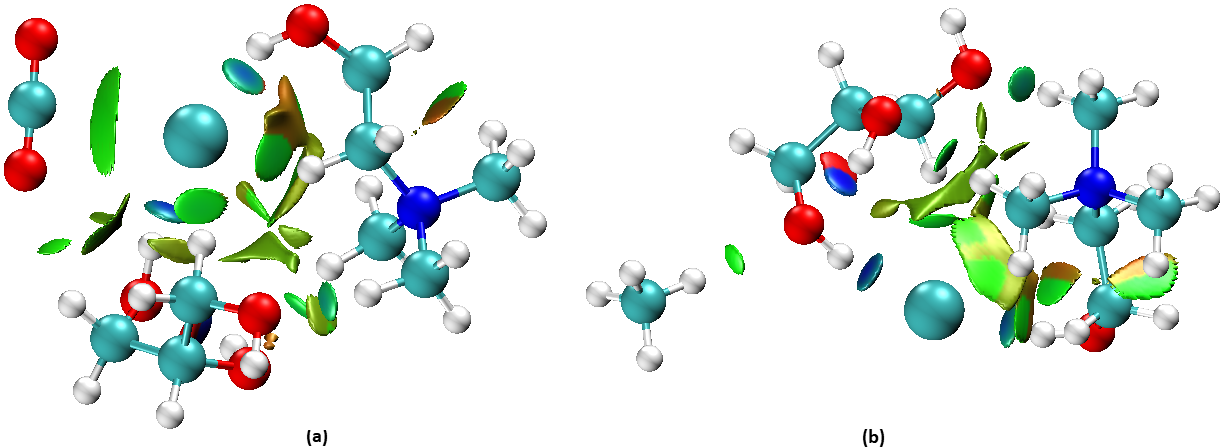


**Figure S8: RDG (0.5) iso-surfaces for (a) DES-CO_2_ and (b) DES-CH_4_ complex**
